# Gender Differences in Clinical Practice Regarding Coronary Heart Disease: A Systematic Review

**DOI:** 10.3390/jcm14051583

**Published:** 2025-02-26

**Authors:** Emily Caitlin Lily Knox, Inmaculada Mateo-Rodríguez, Antonio Daponte-Codina, Fernando Rosell-Ortiz, Silvia Solá-Muñoz, Antía Codina-Rodríguez, Héctor Bueno, José Ignacio Ruiz-Azpiazu

**Affiliations:** 1 Andalusian School of Public Health, 18011 Granada, Spain; emily_knox2@hotmail.co.uk (E.C.L.K.); adapontec@gmail.com (A.D.-C.); 2 CIBER Epidemiology and Public Health (CIBERESP), 28029 Madrid, Spain; 3 Facultad de Psicología, Universidad Nacional a Distancia (UNED), 28015 Madrid, Spain; 4 Servicio de Emergencias 061 de La Rioja, Centro de Investigación Biomédica de La Rioja (CIBIR), 26008 Logroño, Spain; fernandorosell@gmail.com (F.R.-O.); jirazpiazu@riojasalud.es (J.I.R.-A.); 5 Sistema de Emergencies Mediques de Catalunya, 08908 Hospitalet de Llobregat, Spain; silviasola@gencat.cat; 6 Institut d’Investigació Sanitaria Pere i Virgili (IISPV), 43007 Tarragona, Spain; 7 Centro de Salud Universitario Goya, Hospital La Princesa, 28006 Madrid, Spain; antia.codina@salud.madrid.org; 8 Centro Nacional de Investigaciones Cardiovasculares (CNIC), 28029 Madrid, Spain; hector.bueno@cnic.es; 9 Cardiology Department, Hospital Universitario 12 de Octubre and Instituto de Investigación Sanitaria Hospital 12 de Octubre, 28041 Madrid, Spain; 10 Centro de Investigación Biomédica en Red Enfermedades Cardiovaculares (CIBERCV), 28029 Madrid, Spain; 11 Facultad de Medicina, Universidad Complutense de Madrid, 28040 Madrid, Spain

**Keywords:** coronary heart disease, gender, treatments, review, cardiovascular, women

## Abstract

**Background/Objectives**: A systematic review was performed with the aim of analysing potential sex differences in the overall treatment of coronary heart disease (CHD). **Methods**: Studies published between January 2011 and November 2023 that conducted a sex-based analysis of the provision of any type of therapeutic measure to treat CHD were included. A search was performed of the Web of Science database in November 2023, resulting in 9070 articles. Study quality was examined using the Newcastle–Ottawa scale. A worksheet was produced to extract data pertaining to the title, year of publication, sample, context, study design, dependent variables, time-frame, treatment type, and outcomes reported by each article. This systematic review followed PRISMA guidelines, and the research protocol was submitted to PROSPERO (CRD42022330238). **Results**: A total of 80 articles presenting data representing 560.070,624 individual datapoints were selected to comprise the final sample. The main findings revealed that the majority of studies highlighted inequalities that disadvantaged females in all analysed treatment categories (pharmacological treatment, invasive interventions, rehabilitation programmes, and other treatment types). **Conclusions**: Despite the abundance of evidence on the need to improve healthcare provision to females with CHD, few studies examined the reasons or mechanisms underlying the inequalities identified.

## 1. Introduction

Cardiovascular diseases (CVDs), led by coronary heart disease (CHD), are one of the leading causes of death worldwide [1]. Despite females exhibiting a lower incidence of such diseases, studies demonstrate that they suffer from a higher mortality rate and worse prognosis than males following an acute cardiovascular event [2,3]. In fact, CVDs are responsible for 487,209 male (21.1% to 28.4% depending on ethnic/racial subgroup) and 441,532 female deaths (23.4% to 28.9% depending on ethnic/racial subgroup) in the US and 1.8 million male (39%) and 2.1 million female deaths (46%) in Europe. For this reason, it is highlighted as the main cause of death amongst women [4].

Despite the huge impact of CVD on the female population, CVD continues to be considered a ‘male issue’ in many contexts, especially when it comes to CHD [5]. This perception has driven what has been denominated by Woodward as ‘the female disadvantage’ with regard to CVD [6]. The female disadvantage outlines three dimensions in which the existence of a gender gap may explain potential overall differences between males and females in CVD.

The first dimension explores risk factors. It describes the way in which aspects such as the lower participation of females in clinical trials [7] has impeded research into the modifying effect of biological factors on the impact of health-related behaviours (e.g., smoking, physical activity) and other factors (e.g., hypertension, excess body weight). Such aspects may lead to many potential prevention measures being less effective in females.

The second dimension is related to individual knowledge and behaviour in relation to CVD. Females perceive themselves to be less at risk of suffering from CVD, and this lower awareness of their own risk may drive delays in decisions to seek healthcare [8].

The third and final dimension describes the differential responses of professionals. It highlights differences in the healthcare provision on offer and delivered to patients as a function of sex. Potential causes of such differences may be related to the fact that, within professionals, there is a prevailing belief that CVDs are, fundamentally, a male issue, with regard to prevalence, symptoms, and treatment effectiveness.

Coronary heart disease is the main form of CVD. In the classical definition conceived by Heberden more than 200 years ago, diagnosed cases were essentially in males due to the life expectancy at the time and the later presentation of the disease in females [9]. It is surprising that, in the present day, this bias remains in clinical guidelines [10]. For this reason, research into the avoidable causes of gender bias in CHD is essential.

The present study focuses on the last of the three dimensions of ‘female disadvantage’, specifically the dimension pertaining to healthcare provision by healthcare professionals. A systematic review was performed to examine potential sex differences in the treatment of CHD. The aim of this was to identify any potential gaps in healthcare provision to females diagnosed with CHD from disease prevention to rehabilitation.

## 2. Materials and Methods

The present systematic review was performed in accordance with the PRISMA 2020 checklist [11] and was registered in PROSPERO on the 15th of May 2022 with the identification number CRD42022330238.

Firstly, a search was conducted of the Web of Science database in November 2023. The search was restricted by publication year, including articles published between January 2011 and November 2023. The following keywords, alongside the relevant Boolean operators, were employed: ‘acute coronary syndrome’; ‘coronary artery disease’; ‘coronary heart disease’; ‘myocardial infarction’; ‘ischemic heart disease’; ‘gender’; ‘sex’; ‘treatment’; ‘medication’; ‘stent’; ‘drug’; ‘therapy’; ‘pharmaco*’; ‘prescription’; and ‘intervention’. The initial search recovered a total of 9070 records.

Next, a preliminary review was performed of the titles and abstracts of the 9070 gathered records in order to evaluate whether they were suitable for inclusion in the subsequent stage of the selection process. For this, the following inclusion criteria were established: (1) studies performed with samples of adults (older than 17 years of age) from the general population diagnosed with a CVD with ICD10 codes I20–I25 (ischaemic heart diseases); (2) the inclusion of a dependent variable that represented the performance of some type of treatment measure (e.g., medication use, surgical procedures, referral or attendance to rehabilitation programmes, fulfilment of healthcare guidelines pertaining to CHD, amongst others); and (3) studies that conducted a comparative analysis of main variables as a function of sex. On the other hand, articles that met one or more of the following exclusion criteria were excluded: (1) the application of strict criteria to limit participant selection based on age or include a specific sub-population of adults, such as studies only including adults older than 70 years; (2) failure to specify the specific CHD diagnosis; and (3) the presentation of separate findings for males and females without comparative analysis.

Of the 9.070 titles and abstracts reviewed, 244 were selected to proceed to the next stage of analysis. Further, 35 literature reviews were identified (amongst them were short reviews, narrative reviews, systematic reviews, and meta-analyses).

In the next stage, the full texts of the 244 articles identified in the previous stage were reviewed. Of these, 87 articles were discarded due to not conducting analysis according to sex (*n* = 11), not performing a relevant comparison (*n* = 2), not including diagnosed patients (*n* = 3), not including a sample of adults from the general population (*n* = 3), including adults diagnosed according to ICD codes not covered by the inclusion criteria (*n* = 15), not administering a relevant treatment (*n* = 3), not evaluating a relevant outcome variable (*n* = 37), only having a full text available in Portuguese (*n* = 1), only presenting qualitative outcomes (*n* = 2), not performing analysis according to sex in addition to not evaluating a relevant outcome variable (*n* = 5), and not providing access to the full text (*n* = 5).

Next, the reference lists of the 35 reviews identified in the initial search were perused to confirm whether any further relevant studies could be retrieved. From these reviews, 30 further articles were identified. Of these, five articles were discarded, following full-text review, due to not including a relevant dependent variable (*n* = 2) and not comparing males and females (*n* = 3). Finally, 25 articles were carried forward for further analysis together with the 157 articles identified in the previous stage, leaving an overall sample of 181 articles.

One researcher read the full texts of all articles in depth and gathered/recorded data pertaining to the article title, authors, year of publication, sample, context, research design, variables, follow-up/intervention time-frame, administered/observed treatment, main findings, conclusions, and explication of/justification for sex differences. All data were recorded in three tables that were organised according to treatment type in order to enable separate analysis of the use of pharmacological treatment, invasive interventions/surgeries (for example, stents, PCI, bypass grafts), rehabilitation programmes, and other treatments.

The tables produced and the articles included in this review were reviewed by another five authors in order to verify conclusions regarding the contributions made by each article and the veracity of the extracted data and conclusions presented in the tables. Overall final outcomes were discussed by all reviewers as a group.

### Study Quality

The Newcastle–Ottawa Quality Index [12], adapted for use with multiple-measure cohort studies or cross-sectional studies, was used, where relevant, to analyse and organise studies according to methodological quality (Tables S1 and S2 of the Supplementary Materials). The analysis of study quality revealed that 80 (43.6%) studies were of high quality, 13 (7.2%) were of average quality, and 89 (49.2%) were of low quality. The reason for the low-quality rating, in the majority of cases (i.e., for 74 of the 89 low-quality studies), was that the analysis failed to control for potentially important confounding factors such as age, race, and socioeconomic status. Other reasons were that the study failed to fully describe the statistical test used or failed to report all outcomes including *p*-values and confidence intervals. The decision was taken to exclude low- and average-quality studies and only proceed with further analysis of the 80 high-quality studies.

As a means of facilitating the interpretation of outcomes and to be able to draw useful conclusions from the present review, findings were organised according to four groups (Table 1, Table 2 and Table 3), according to the type of intervention or treatment applied, i.e., pharmacological treatments, invasive therapies, rehabilitation programmes, and other actions or aspects that influence cardiovascular outcomes.

## 3. Results

A total of 80 articles comprised the final sample, of which 77 presented data on 16,169,348 patients and three presented data on 543,904,382 clinic visits.

**Figure 1:**
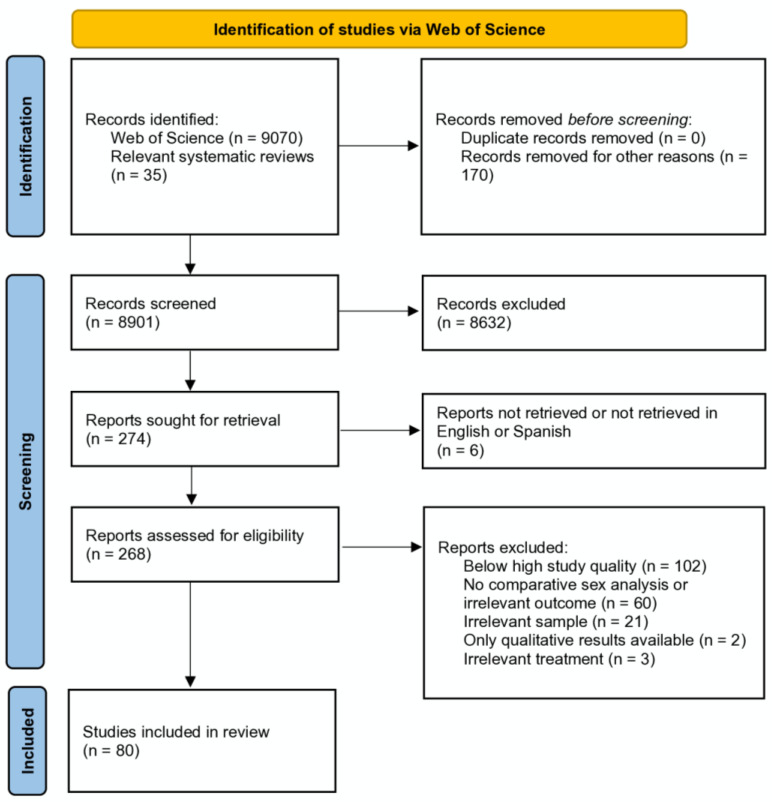
Study flow diagram.

A study flow diagram is presented in Figure 1.

### 3.1. Use of Pharmacological Treatment

A total of 39 articles were identified that examined the use of pharmacological treatment in the CHD process (acute and chronic treatment and primary and secondary prevention) (Table 1). Of these, 33 (84.6%) demonstrated a lower use of one or more pharmacological treatments, less appropriate treatment according to international recommendations, or less guidance around the use of pharmacological treatment in women relative to men. In twenty-four of these studies, the use of one or more specific pharmacological treatments was found to be less common in females than in males (statins = sixteen; aspirin = five; antiplatelets = four; beta blockers = three; ACE inhibitors = one; thrombolytics = one; thienopyridines = one; antiplatelet receptor blockers = two; lipid-lowering drugs = three; clopidogrel or other P2y12 inhibitors = two; heparin = two; and glycoprotein IIb/IIIa inhibitors = one) [13,14,15,16,17,18,19,20,21,22,23,24,25,26,27,28,29,30,31,32,33,34,35]. Three articles revealed that female sex was a predictor of not receiving or not adhering to an indicated prescription according to guidelines outlined by the European Society of Cardiology (ESC) or the American College of Cardiology/American Heart Association (ACCF/AHA) in relation to aspirin, statins, double antiplatelet therapy with a P2y12 inhibitor, beta blockers, and ACE inhibitors [36,37,38]; two articles found that fewer females received DAPT [16,39]; one article revealed female sex to be a predictor of not receiving any type of medical prescription [40]; eight articles reported less intense or more conservative use of pharmacological drugs in females [18,26,32,33,41,42,43,44]; and five articles concluded that the discontinuation of treatment or poor adherence was more likely in females [18,32,42,43,45]. In contrast, a total of one (2.6%) article reported greater use of pharmacological treatment in females than in males. This article [46] reported that more females were medicated with a lipid-lowering drug. Finally, four (10.3%) articles did not find any significant differences between males and females in the use of pharmacological treatment [47,48,49,50], whilst one (2.6%) study found some pharmacological drugs to be used more by males and others to be used more by females [51].

**Table 1:** Characteristics of studies included in this review—pharmacological treatment.

Author	Title	Year	Sample	Context	Design	Time-Frame	Treatment	Main Findings
[13]	Statin use in outpatients with obstructive coronary artery disease	2011	38,775 patients with obstructive CVD	The US between 1 July 2009 and 30 June 2010	Longitudinal registry PINNACLE		Statins and other medication	Male sex was a predictor of statin use.
[14]	Bridging the gender gap: Insights from a contemporary analysis of sex-related differe…	2012	14,196 ACS STEMI patients	Data from four Canadian registries between 1999 and 2008	Prospective, multi-centre, and observational		Aspirin, ticlopidine/clopidogrel, heparin, and GPIIb/IIIa inhibitors	Fewer women received thienopyridines, heparin, or glycoprotein IIb/IIIa inhibitors.
[15]	Statin prescription for patients with atherosclerotic cardiovascular disease from *n*…	2019	8468 CVD patients (29.3% women)	Two national surveys between 2011 and 2015 in the US	Cross-sectional and retrospective		Statins	Men were more likely to receive a statin prescription. The effect persisted after adjusting for those with coronary disease.
[16]	Sex differences in in-hospital management and outcomes of patients with acute coronary syndrome	2019	82,196 ACS patients from 192 hospitals	AHA and Chinese Society of Cardiology national registry between 2014 and 2018	Prospective and observational		ACE inhibitors/ARBs, beta blockers, statins, and heparin	Fewer women received acute treatment based on scientific evidence, anti-platelet therapy, enzyme inhibitors, and statins. The likelihood of receiving pharmacological treatment was greater in men than in women in the over-65s. In the under-65s, men received more thromolytic drugs than women.
[17]	Temporal trends in the incidence, treatment patterns, and outcomes of coronary artery disease and peripheral …	2020	231,120 ACS patients	4.6 million individuals from the UK between 2006 and 2015	National, representative, observational digital registries		Statins	Fewer statins were prescribed in women.
[18]	Trends in use of high-intensity statin therapy after myocardial infarction, 2011 to 2014	2017	42,893 MarketScan patients and 75,096 Medicare patients	Health insurance claimants after suffering CHD, the US, 2011 to 2014	Observational	Hospital discharge	Statins	More men received high-intensity statin prescriptions than women.
[19]	National trends and disparities in statin use for ischemic heart disease from 2006 to 2018: Insights from National Ambul…	2022	542,704,112 outpatient CHD appointments	National Ambulatory Medical Care Survey (NAMCS), the US, Jan 2006 to Dec 2018	Retrospective analysis	During outpatient appointment	Statins	Male sex was associated with a greater likelihood of statin use.
[20]	Treatment gaps, 1-year readmission and mortality following myocardial infarction by diabetes status, sex and so…	2022	43,272 patients after MI	All MI survivors in Victoria, Australia, Jul 2012-Jun 2017	Observational cohort	One year	Cardioprotective medications	Males were more likely to be dispensed cardioprotective medications at or within 90 days of discharge.
[21]	Sex differences in time trends in acute coronary syndrome management and in 12-month lethality: Data from the Frenc…	2022	3196 patients with first ACS	All patients (aged 35–74) form MONICA registries in France, Jan-Dec 2006 or Oct 2015-March 2016	Observational cohort	Twelve months in 2006 or six months in 2016	Beta blockers, platelet aggregation inhibitors, statins, ACE inhibitors, and anticoagulants	Prescriptions of platelet aggregation inhibitors and statins were still more frequently provided to men than to women in 2016, independently of confounders.
[22]	Sex differences in characteristics, treatments, and outcomes among patients hospitalized for non–ST-segm…	2022	4611 NSTEMI patients (39.8% women)	151 Chinese hospitals, nationally representative of patients admitted in 2006, 2011, and 2015	2-stage random sampling design	In hospital	Aspirin, clopidogrel/ticagrelor, dual antiplatelet therapy, β-blockers, ACE inhibitor/ARB, statins, and parenteral anticoagulant	Women were less likely to receive treatments than men, with significant differences for aspirin in 2015.
[23]	Trends in provision of medications and lifestyle counseling in ambulatory settings by gender and race f…	2023	11,033 appointments for adults with atherosclerotic cardiovascular disease	Nationally representative ambulatory care, the US, 2006–2016	Cross-sectional	In clinic	Statin therapy and aspirin prescription	Women were less likely than men to receive statin therapy and aspirin prescription.
[24]	Population-wide cohort study of statin use for the secondary cardiovascular disease prevention in Scotland in 20…	2022	167,978 patients with an ASCVD event	Linked National Health Service Scotland administrative data, 2009–2017	National retrospective cohort	Average: 4.6 years	Statins	Women were less likely to initiate therapy.
[25]	Persisting gender differences and attenuating age differences in cardiovascular drug use for prevention and t…	2013	1,230,290 patients from primary care and 15,651 patients from secondary care	PHARMO database between 1998 and 2010, Holland	Cohort and observational	36 months	Lipid-lowering drugs, blood-pressure lowering drugs, beta blockers, and antithrombotics	In primary care, fewer women used lipid-lowering drugs. During hospitalisation, fewer women used cardiovascular drugs. In secondary care (36 months after discharge), young women used fewer drugs in general.
[26]	Age and gender differences in medical adherence after myocardial infarction: Wome…	2018	59,534 patients (34% women)	Dutch health insurance register from 2012 and 2013	Retrospective, cohort, and observational	One year	Aspirin, P2y_12_-inhibitors, statin, beta blockers, ACE-/AT2-inhibitors, vitamin K antagonists, novel oral anticoagulant	There was lower use of five indicated drug treatments in women; differences were more evident in those with NSTEMI.
[27]	Dyslipidemia management in patients with coronary artery disease. Data from the POLASPIRE survey	2021	1236 CHD patients (29% women)	Poland, within the prior 6 to 24 months	Cross-sectional and multi-centre	Six–eighteen months after hospital discharge	Statins and other medications	Male sex was correlated with statin prescriptions at discharge.
[28]	Drivers of the sex disparity in statin therapy in patients with coronary artery disease: A cohort study	2016	24,338 CHD patients (9006 [37%] women)	Between 2000 and 2011 at two academic medical centres in Boston	Retrospective cohort	Followed for at least one year	Statins	Women were less likely to either have initiated statin therapy or to have persistent statin therapy at the end of follow-up.
[29]	Tackling inequalities: are secondary prevention therapies for reducing post-in…	2014	1327 patients discharged after a heart attack	Municipality of Rovigo, Italy, between January 2002 and November 2009	Retrospective cohort and observational	Average follow-up: 3.39 years	ACE inhibitors, beta blockers, platelet aggregation inhibitors, statins, omega-3 triglycerides, and anticoagulants	Women were less likely to use statins and anti-platelet drugs.
[30]	Age and sex inequalities in the prescription of evidence-based pharmacological therapy follo…	2014	747 STEMI patients and 1364 NSTEMI patients (67% men)	10 Portuguese hospitals, 2008–2009	Consecutive cases and observational	Hospital discharge	Aspirin, clopidogrel, beta blockers, ACE inhibitor/ARB, and statins	There was a lower likelihood of women receiving aspirin and clopidogrel at discharge.
[31]	Sex differences in the treatment and outcome of Korean patients with acute m…	2015	85,329 MI patients	Korean hospitals between 2003 and 2007	Retrospective and observational cohort	30 days	Aspirin, thrombolytic drugs, beta blockers, and cholesterol-lowering drugs	After adjustment, men were more likely to receive pharmacological treatments.
[32]	Investigating the prevalence, predictors, and prognosis of suboptimal statin use early af…	2017	1005 ACS NSTEMI patients	16 hospitals in the UK between 2008 and 2013	Prospective and cohort	Median follow-up: 16 months	Intensive statin therapy	Female sex was a predictor of the underuse of optimal statin treatment (reduced doses, cessation, changing to a less potent statin, non-compliance with treatment).
[33]	Predictors of nonuse of a high-potency statin after an acute coronary syndrome: Insights from the stabilization of plaq…	2017	12,446 ACS patients	36 countries between 2009 and 2011	Retrospective analysis of data from a double-blind, controlled, and randomised study	Three months	Statins and high-intensity statins	Female sex predicted the non-use of statins. Patients not treated with high-intensity statins were more likely to be women.
[34]	Differences in management and outcomes for men and women with ST-elevation myocardial infarction	2018	2898 STEMI patients (715 women)	Registry of 41 Australian hospitals between February 2009 and May 2016	Cohort, prospective, and observational	Six months	Aspirin, second antiplatelet, beta blockers, ACE inhibitor/ARB, and statins	At discharge, fewer women received beta blockers and statins.
[35]	Gender-related differences in antiplatelet therapy and impact on 1-year clinical outc…	2019	840 ACS patients (26% women)	8 centres between January 2014 and December 2016	Multi-centre registry and observational	Follow-up: one year	Dual anti-platelet therapy, ticagrelor, clopidogrel, and Prasugrel	Drug-eluting stents and dual anti-platelet therapy were used more in men than in women.
[36]	Trends in gender differences in cardiac care and outcome after acute myocardial infarction in western Sweden: A report fro…	2015	48,118 MI patients (35.4% women)	Cardiac units in Sweden between January 1995 and October 2014	National registry and observational	In hospital, at thirty days and at one year	Acetylsalicylic acid, aniplatelets, beta blockers, ACE inhibitor/ARB, oral anticoagulants, statins, IV diuretics, and IV inotropes	Compared to men, women younger than 60 were less likely to receive indicated treatment at discharge (beta blockers, ECAn or angiotensin receptor inhibitors, statins, and P2y12 antagonists).
[37]	Long-term quality of prescription for ST-segment elevation myocardial infarction (STEMI) patients: A real world…	2020	361 STEMI patients	A hospital in Switzerland between 2014 and 2016	Prospective and observational	At discharge and after one year	Aspirin, P2y12 inhibitors, statin, ACEn inhibitors, and beta blockers	Female sex was a predictor of not receiving an appropriate prescription at discharge, but it was not a predictor after one year.
[38]	Gender difference in secondary prevention of cardiovascular disease and outcomes following the survival of acut…	2021	9283 discharged ACS patients	43 Australian hospitals between 2009 and 2018	Observational and prospective	Six and thirteen months	Aspirin, lipid-lowering therapy, anti-platelet drugs, beta blockers, and enzyme inhibitors	After one year, women were less likely to be taking more than 75% of examined medications.
[39]	Sex differences in distribution, management and outcomes of combined ischemic-bleeding …	2021	584,360 ACS patients	Hospitals in the UK between January 2010 and December 2017	Retrospective		ACE inhibitors, beta blockers, statins, antiplatelets, aspirin, P2y12 inhibitor, and DAPT	In high-risk patients, women were less likely to receive guideline-recommended treatment (dual anti-platelet therapy).
[40]	Sex disparities in post-acute myocardial infarction pharmacologic treatment init…	2015	12,261 MI survivors (3783 women)	Registries from British Colombia between 2007 and 2009	Retrospective, populational, and cohort	One year after discharge	Prescriptions for ACEn inhibitors, beta blockers, and statins	Young men were more likely than women to start treatment. There were no sex differences in treatment compliance.
[41]	Clinical implications of switching from intensive to moderate statin therapy after acute coronary syndromes	2011	1321 CHD patients (886 men)	All patients discharged from an Italian unit after a CHD in a 6.5-year period	Cohort and observational	12 months	Intensive versus moderate statins	Statin therapy was more likely to be changed from a high intensity to a moderate intensity in women.
[42]	Sex differences in treatment and prognosis of acute coronary syndrome with inter…	2018	1214 patients (24% women)	One Spanish centre, January 2013 to January 2016	Prospective, consecutive, cohort, and observational		Ticagrelor/Prasugrel, ACE inhibitors, beta blockers, and statins	Fewer women received potent anti-platelet drugs.
[43]	Prasugrel for Japanese patients with ischemic heart disease in long-term clinical practice (PR…	2020	4155 patients treated for CVD	Japan between 1 June 2015 and 31 May 2018	Observational	One year	Prasugrel and aspirin	Female sex predicted that treatment was employed for less than 360 days.
[44]	Gender disparities in evidence-based statin therapy in patients with cardiovascular disease	2015	972,532 CVD patients	130 US healthcare units for veterans between October 2010 and September 2011	National cohort and observational		Statins	Women were less likely to receive statins or high-intensity statins. Female sex was an independent predictor of not receiving statins or high-intensity statins, with significant variability as a function of the unit.
[45]	Incidence, predictors, and clinical impact of early prasugrel cessation in patients with ST-elevation myocardial …	2018	1830 patients (17% women) treated with stents	All patients at Bern University Hospital, Switzerland, in 2009	Two prospective registries and a clinical trial	One year	Prasugrel after stenting	Female sex predicted the discontinuation or interruption of treatment and cessation of Prasugrel for any reason.
[46]	No gender differences in prognosis and preventive treatment in patients with A…	2012	1595 patients (834 women) hospitalised with MI with no significant stenoses found in coronarography	Danish registers between 2005 and 2007	National cohort	60 days after discharge	Lipid inhibitors, beta blockers, cloridogrel, and aspirin	More women took lipid inhibitors; there were no other sex differences.
[47]	Medical care of acute myocardial infarction patients in a resource limiting country,…	2019	1106 patients (67.3% men)	A hospital in Trinidad between March 2011 and March 2015	Retrospective, cross-sectional, and observational		Different medications and therapies	No sex difference was found in medication use, although there were few data for women.
[48]	Sex differences in cardiac medication use post-catheterization in patients undergoing coronary angiography	2017	All angina patients treated with angiography in British Columbia (*n* = 7.535)	British Columbia between January 2008 and March 2010	Observational	Three months prior to and three months after treatment	ACEn inhibitors, angiotensin receptor blockers, calcium channel blockers, beta blockers, statins, and anti-platelet agents	There were no significant differences according to sex.
[49]	Propensity score-matched analysis of effects of clinical characteristics and treatment…	2011	3510 consecutive patients (32% women) after MI	10 cardiology centres in eastern France between January 2006 and December 2007	Observational and two paired cohorts	30 days	Aspirin, clopidogrel, ACE inhibitor/ARB, beta blockers, and statins	Women received less medical treatment. After adjustment, this effect disappeared.
[50]	Medical treatment in coronary patients: Is there still a gender gap? Results from European S…	2020	8261 patients (25.8% women)	ESC EORP EUROASPIRE V survey of 131 centres in 27 countries, 2017–2017	Cross-sectional and observational	Six months and two years	Aspirin/other antiplatelets, beta blockers, ACE inhibitors/ARB, ACE-I, ARB, statins, anticoagulants, calcium channel blockers, diuretics, glucose-lowering drugs, insulin, antidepressants, and anti-anxiety drugs	Generally, there were no sex differences in the number of prescriptions or medication use. Fewer women were prescribed statins but more women were prescribed diuretics.
[51]	Sex differences in the treatment and outcome of patients with acute coronary …	2014	32,821 ACS patients (8884 women) treated with PCI with ischemic bleeding risk	Taiwan between January 2006 and December 2007	Retrospective cohort and observational	Follow-up: minimum of one year	Aspirin, clopidogrel, beta blockers, ACE inhibitor/ARB, and statins	Women were less likely to receive aspirin and clopidogrel but more likely to receive beta blockers and statins.

Note: CVD: cardiovascular disease; CHD: coronary heart disease; ACS: acute coronary syndrome; MI: myocardial infarction; PCI: percutaneous coronary intervention; STEMI: ST-elevation myocardial infarction; NSTEMI: non-ST-elevation myocardial infarction; ACE: angiotensin-converting enzyme.

### 3.2. Invasive Treatments, Percutaneous Coronary Interventions (PCIs) or Surgery, and Thrombolytic Reperfusion Therapy in Both Acute and Chronic Stages

Next, 38 articles were identified that examined the use of invasive treatments, percutaneous coronary interventions (PCIs) or surgery, and thrombolytic reperfusion therapy in both acute and chronic stages (Table 2). Of these, 36 (94.7%) studies concluded that females were either less likely to receive some type of invasive therapy or had to wait longer to receive this type of treatment. Of the articles reflecting inequality that was disadvantageous towards females, 31 articles reported that females were less likely to receive one or more types of invasive therapy [16,20,21,22,31,34,35,36,39,47,49,52,53,54,55,56,57,58,59,60,61,62,63,64,65,66,67,68,69,70,71]. Four articles highlighted that females experienced longer delays prior to receiving an invasive therapy [72,73,74,75], whilst one article revealed that females were less likely to be referred to a PCI centre [63] and another article reported that females were more likely to receive inappropriate treatment [76]. In contrast, one article (2.7%) found that females were more likely than males to receive invasive therapy, namely PCI with or without stenting in STEMI [77], whilst another study (2.7%) concluded that no differences emerged as a function of sex [78].

**Table 2:** Characteristics of studies included in this review—invasive interventions.

Author	Title	Year	Sample	Context	Design	Time-Frame	Treatment	Main Findings
[16]	Sex differences in in-hospital management and outcomes of patients with acute coronary syndrome	2019	82,196 ACS patients from 192 hospitals	AHA and the Chinese Society of Cardiology national registry between 2014 and 2018	Prospective and observational		Reperfusion therapy	Women were less likely to have received reperfusion treatment for STEMI, including PCI.
[20]	Treatment gaps, 1-year readmission and mortality following myocardial infarction by diabetes status, sex and so…	2022	43,272 patients after MI	All survivors of MI in Victoria, Australia, July 2012–June 2017	Observational cohort	One year	PCI and CABG	Females were less likely to receive either PCI or CABG.
[21]	Sex differences in time trends in acute coronary syndrome management and in 12-month lethality: Data from the Frenc…	2022	3196 ACS patients	All patients (aged 35–74) from the MONICA registries, France, January–December 2006 or October 2015–March 2016	Observational cohort	Twelve months in 2006 or six months in 2016	Revascularisation	Revascularisation treatment was still more frequently provided to men than to women in 2016, independently of confounders.
[22]	Sex differences in characteristics, treatments, and outcomes among patients hospitalized for non–ST-segm…	2022	4611 NSTEMI patients (39.8% women)	151 Chinese hospitals with patients admitted in 2006, 2011, and 2015	Two-stage random sampling design	In hospital	PCI and other invasive strategies	Women were less likely to receive treatments than men, with significant differences for PCI and invasive strategies in 2011 and 2015.
[31]	Sex differences in the treatment and outcome of Korean patients with acute m…	2015	85,329 MI patients	Korean hospitals between 2003 and 2007	Retrospective, cohort, and observational	30 days	Coronary angiography, cardiac catheterisation, PCI, and CABG	After adjustment, fewer women than men received catheterisation, grafts, and PCI.
[34]	Differences in management and outcomes for men and women with ST-elevation myocardial infarction	2018	2898 STEMI patients (715 women)	A registry of 41 Australian hospitals between February 2009 and May 2016	Cohort, prospective, and observational	Follow-up: six months	Coronary angiography, total revascularisation, timely revascularisation, PCI, thrombolysis, and CABG	Women were less likely to have received treatment with PCI or CABG, coronary angiography, or revascularisation.
[35]	Gender-related differences in antiplatelet therapy and impact on 1-year clinical outc…	2019	840 ACS patients (26% women)	Eight Italian centres between January 2014 and December 2016	Multi-centre registry and observational	One year	Drug-eluting stents	Drug-eluting stents were used more in men than in women.
[36]	Trends in gender differences in cardiac care and outcome after acute myocardial infarction in western Sweden: A report fro…	2015	48,118 MI patients (35.4% women)	Cardiac units in Sweden between January 1995 and October 2014	National registry and observational	In hospital, after thirty days and one year	Coronary angiography and PCI (reperfusion treatment)	Women with STEMI were less likely to undergo coronary angiography than men with STEMI in adjusted analysis.
[39]	Sex differences in distribution, management and outcomes of combined ischemic-bleeding …	2021	584,360 ACS patients	Hospitals in the UK between January 2010 and December 2017	Retrospective		Guideline-recommended therapies	In high-risk patients, women were less likely to receive guideline-recommended therapies (revascularisation, PCI, and graft).
[47]	Medical care of acute myocardial infarction patients in a resource limiting country,…	2019	1106 patients (67.3% men)	One hospital in Trinidad between March 2011 and March 2015	Retrospective, cross-sectional, and observational		Therapies, surgery, and thrombolysis	Generally, surgery was reported to be little used. More men were treated with thrombolysis.
[49]	Propensity score-matched analysis of effects of clinical characteristics and treatment…	2011	3510 patients (32% women) after MI	Consecutive cases between January 2006 and December 2007 from 10 cardiology centres in eastern France	Observational and two matched cohorts	30 days	PCI, CABG, thrombolysis, and other reperfusion	Fewer women were treated with angiography and reperfusion, even after adjustment.
[52]	Does prior coronary artery bypass surgery alter the gender gap in patients presenting wit…	2012	16,750 ACS patients	The registry of a hospital in Doha, Qatar, between January 1991 and December 2010	Retrospective, observational, and cohort		Thrombolysis, PCI, and reperfusion	Women were less likely to receive reperfusion and early invasive therapies.
[53]	Disparities by race, ethnicity, and sex in treating acute coronary syndromes	2012	20,604 Medicare claimants (health insurance) with ACS	A US registry in 2001	Retrospective and observational		Coronary revascularisation	Women presented lower rates than men of any type of revascularisation. Black women had the lowest rates.
[54]	Do clinical factors explain persistent sex disparities in the use of acute reperfusion ther…	2013	32,676 STEMI patients	Sweden between 2004 and 2008	Retrospective		Reperfusion therapy	Reperfusion therapy used less in women; reasons varied as a function of age.
[55]	Gender, socioeconomic position, revascularization procedures and mortality in patients presenting with STE…	2014	5792 MI patients	A database from Piedmont, Italy, between January 2008 and December 2008	Observational	In hospital and after one year	PCI	Women were less likely to receive revascularisation.
[56]	Clinical profile of patients with no-reperfusion therapy in Bosnia and Herzegovina and …	2014	633 STEMI patients	14 hospitals in Bosnia and Serbia between October 2012 and September 2013	Observational registry		Reperfusion	Female sex was an independent predictor of reperfusion.
[57]	Gender difference in treatment and mortality of patients with ST-segment elevation myocardial infarction admitte…	2015	8404 STEMI patients	A government registry of 136 hospitals in Victoria between July 2005 and June 2010	Retrospective	Five years	Angioplasty, stent, and CABG	Proportionately fewer women received reperfusion treatment for STEMI.
[58]	Gender differences in the prevalence and treatment of coronary chronic total occlusions	2016	1690 patients with chronic total occlusion and 7682 control	Three units in Canada between Mar 2008 and June 2009	Retrospective, Canadian registry and multi-centre		Revascularisation	The PCI rate in patients was similar between men and women. Fewer women received a bypass graft and, of those receiving a bypass graft, fewer women received revascularisation.
[59]	Revascularization treatment of emergency patients with acute ST-segment elevation myocardial infarction in Switz…	2016	9696 cases (71.6% received revascularisation; 29.2% women)	A Swiss national registry, 300 hospitals, 2010–2011	Observational		Revascularisation	Female sex was a predictor of not receiving revascularisation.
[60]	Reperfusion therapy for ST-elevation acute myocardial infarction in Eastern Europe: the ISACS-TC registry	2016	7982 STEMI patients	57 hospitals in Eastern European countries between January 2010 and February 2015	Observational	30 days	Reperfusion compared with no reperfusion	Female sex was the strongest predictor of no reperfusion.
[61]	Gender based differences in drug eluting stent implantation—data from the German ALKK…	2017	100,704 cases	A national registry of 28 centres in Germany between 2005 and 2009	Prospective and observational		Drug-eluting stents	There were lower rates of drug-eluting stent use in women; this difference was significant in the over-70s.
[62]	Sex-related inequalities in management of patients with acute coronary syndrome—r…	2018	1757 discharged ACS NSTEMI patients and 1184 STEMI patients	10 Portuguese hospitals between 2008 and 2010	Consecutive cases, retrospective, and observational		Revascularisation and reperfusion	In STEMI patients, women were less likely to receive an angiography, even after adjustment, and less likely to receive revascularisation, but not after adjustment.
[63]	Sex, race, and insurance status differences in hospital treatment and outcomes foll…	2018	38,163 OHCA patients (42% women)	The Californian regional registry between 2011 and 2015	Retrospective	Five years	Treatment in a specialised PCI unit	Female sex was a predictor of lower rates of treatment at a PCI unit and with catheterisation.
[64]	Usage of PCI and long-term cardiovascular risk in post-myocardial infarction patient…	2019	32,909 MI patients	Finland between 2009 and 2012	Observational, retrospective cohort	Minimum of one year	PCI	Female sex was an independent predictor of not receiving PCI.
[65]	Healthcare disparities for women hospitalized with myocardial infarction and angina	2020	7878 patients hospitalised due to MI or angina (40% women)	The national Scottish registry between 1 October 2013 and 30 June 2016	Registry and observational	At discharge, six months, and one year	PCI and other coronary angiography	Women were more likely to receive guideline-recommended therapy prior to hospitalisation and more likely to receive an invasive intervention upon arrival. Fewer women received an angiography or PCI and guideline-recommended therapy after MI.
[66]	Age-related sex differences in clinical presentation, management, and outcomes i…	2020	15,532 hospitalised ACS patients	Seven registries from the Persian Gulf between 2005 and 2017	Multi-register	One year	Revascularisation	In patients younger than 65, women were less likely than men to receive thrombolytic therapy and PCI.
[67]	Treatment effect of percutaneous coronary intervention in men versus w…	2021	413,500 (30.7% women) STEMI patients	A national database, the US, between January 2016 and December 2018	Observational		Primary PCI	Women were less likely to receive angiography and PCI.
[68]	Age and sex differences and temporal trends in the use of invasive and noninvasive procedures in patients hospi…	2022	1681 men and 1154 women with initial acute MI	All medical centres in central Massachusetts, 2005–2018	Observational	In hospital	Echocardiography, coronary angiography, PCI, and coronary artery bypass graft surgery	Fewer women underwent cardiac catheterisation, PCI, and coronary artery bypass graft surgery.
[69]	Women are less likely to survive AMI presenting with out-of-hospital cardiac arrest: A nationwide study	2022	16,278 acute MI patients (22.7% women)	The MINAP registry, the UK, 2010–2017	Population-based retrospective cohort study	In hospital	Coronary angiography, PCI, coronary artery bypass graft, and myocardial revascularisation	Women were less likely to receive dual antiplatelet therapy, beta blockers, ACE inhibitors, coronary angiography, and PCI. After adjustment, women had greater odds of receiving coronary angiography and coronary artery bypass graft.
[70]	Sex disparities in diagnostic evaluation and revascularization in patients with acute myocardial infarct…	2023	9,259,932 acute MI patients	A national database of all inpatient discharges from nonfederal hospitals in the US, 2005–2019	Observational cohort; 15 years divided into five 3-year periods	In hospital	PCI and CABG	Women received significantly less PCI and CABG than men in all time periods. In STEMI patients but not in NSTEMI patients, these disparities decreased over time. After all adjustments, gender disparities remained significant for all procedures in NSTEM and STEMI patients in the most recent period.
[71]	Comparisons of the uptake and in-hospital outcomes associated with second-gener…	2016	1,129,122 men and 538,835 women	The CathICP registry, the US, between July 2009 and March 2013	Observational, real-life cohort		PCI with second-generation drug-eluting stents	Second-generation stents were used more in men than in women during the first 1.5 years of the study; after this period, no differences emerged.
[72]	Evaluation of gender differences in door-to-balloon time in ST-elevation myocardial infarction	2013	735 STEMI patients	2006–2010; two STEMI PCI centres based in tertiary care hospitals in Adelaide.	Prospectively designed registry	In hospital	Timely care provision and time to balloon	Women experienced delays in code-to-balloon and, thus, DTB time. After multivariate adjustment, independent determinants of DTB time included female gender.
[73]	Association between gender and shortterm outcome in patients with ST elevation my…	2017	1862 STEMI patients	An international registry between September 2011 and October 2013	Multi-centre, randomised, and double-blinded	30 days	Time to procedure	Women experienced greater delays between symptoms and echography and between PCI and angiography following PCI.
[74]	Time to reperfusion in high-risk patients with myocardial infarction undergoing primar…	2019	1340 STEMI patients	18 Portuguese centres between 2011 and 2016	Observational		Reperfusion with PCI	Greater delays were found in women and, resultantly, time to revascularisation was longer in women than in men.
[75]	Sex differences in time to primary percutaneous coronary intervention and ou…	2022	1244 STEMI patients treated with PCI (24% female)	One tertiary referral centre in Australia, 1 January 2010 to 31 December 2019	Cohort of consecutive cases	10 years	PCI	There were longer median times in females and females were less likely to receive FMC-to-ballon times of less than 90 mins. There were significantly longer total and direct STB, FMCTB, and DTB durations for females. There were significant differences in geometric mean times from symptom onset to FMC and table to balloon.
[76]	Revascularization trends in patients with diabetes mellitus and multivessel coronary arte…	2016	29,769 MI patients from 539 hospitals	The US between July 2008 and December 2014	Observational comparison of two treatments		Bypass graft (the most indicated) compared with PCI (the least indicated)	Female sex predicted PCI use over bypass graft.
[77]	Disparities in drug-eluting stent utilization in patients with acute ST-elevation myocardial infarction: An analysis of the …	2022	1,189,237 ± 18,498.93 inpatient visits with STEMI and PCI (>70% men)	2009 to 2018, the US National Inpatient Sample database	Retrospective	In hospital	Stents	Women were more likely to undergo DES implantation.
[78]	Gender differences in patient and system delay for primary percutaneous coronary intervention: current trends i…	2019	4360 patients (967 women)	A Swiss STEMI healthcare network (24 hospitals) between January 2000 and December 2016	Observational	24 h (from symptoms to PCI)	General healthcare and PCI within 24 h after symptom onset	There was no difference in time taken to seek assistance and receive PCI according to sex.

Note: ACS: acute coronary syndrome; MI: myocardial infarction; PCI: percutaneous coronary intervention; STEMI: ST-elevation myocardial infarction; NSTEMI: non-ST-elevation myocardial infarction; ACE: angiotensin-converting enzyme; OHCA: out-of-hospital cardiac arrest; CABG; coronary artery bypass graft; DES: drug-eluting stent.

### 3.3. Rehabilitation Programmes and Other Interventions

A total of 10 articles were identified that examined rehabilitation programmes (Table 3). Of these, seven (70.0%) articles reported that referral, attendance, or completion rates pertaining to programmes were worse in females than in males [16,21,34,79,80,81,82]. In contrast, two (20.0%) articles demonstrated more benefits in females, including better attendance [83] and lower dropout [84]. In one (10.0%) article, no significant differences emerged according to sex [85].

Finally, eleven studies were identified that examined other actions or interventions (Table 3), and seven of these (63.6%) revealed a gender gap that was disadvantageous towards females. Specifically, three studies reported that females had to wait longer before being attended to in hospital [72,86,87], whilst one study revealed that fewer women were referred to a specialist or specialised unit [88], one study found that fewer women received health-promotion information targeted towards patients [89], one reported that fewer women were correctly triaged and had to wait for more time to receive treatment [90], and one study found that women were more likely to receive a do-not-resuscitate order [63]. In contrast, two (16.7%) articles indicated that no significant differences existed according to sex in access to lifestyle counselling [23] and stress testing [68]. Finally, one article revealed that more women received some type of follow-up [91] or pharmacotherapy to help them quit smoking [92].

**Table 3:** Characteristics of studies included in this review—rehabilitation programmes and other.

Author	Title	Year	Sample	Context	Design	Time-Frame	Treatment	Main Findings
*Rehabilitation programmes*								
[16]	Sex differences in in-hospital management and outcomes of patients with acute coronary syndrome	2019	82,196 ACS patients from 192 hospitals	AHA and the Chinese Society of Cardiology national registry between 2014 and 2018	Prospective		Rehabilitation programme	Fewer women received counselling to quit smoking and rehabilitation.
[21]	Sex differences in time trends in acute coronary syndrome management and in 12-month lethality: Data from the Frenc…	2022	3196 patients with first ACS	All patients (aged 35–74) from MONICA registries, France, January–December 2006 or October 2015–March 2016	Observational cohort	Twelve months in 2006 or six months in 2016	Functional rehabilitation	Functional rehabilitation was still more frequently provided to men than to women in 2016, independently of confounders.
[34]	Differences in management and outcomes for men and women with ST-elevation myocardial infarction	2018	2898 STEMI patients (715 women)	A registry of 41 Australian hospitals between February 2009 and May 2016	Cohort, prospective, and observational	Six months	Cardiac rehabilitation referral	At discharge, fewer women were referred to a rehabilitation programme.
[79]	Trends and disparities in referral to cardiac rehabilitation after percutane…	2011	145,661 patients following PCI	31 hospitals in Michigan between 2003 and 2008	Observational registry	Six years	Rehabilitation programme	There was a lower referral rate in women.
[80]	Predictors of cardiac rehabilitation referral, enrolment and completion af…	2021	666 heart attack patients (66% men)	Hospitalisations at a single centre in Holland in 2015 or 2016	Retrospective cohort	Maximum of six months after the event	Rehabilitation programme	Non-referral was associated with the female sex.
[81]	Cardiac rehabilitation attendance and outcomes in coronary artery disease patients	2012	5886 patients (20.8% women) after angiography	Calgary, Canada, between 1996 and 2009	Prospective cohort	Programme start and 12 weeks later	12-week cardiac rehabilitation programme	Women were less likely to finish rehabilitation.
[82]	Predictors of early and late enrollment in cardiac rehabilitation, among those r…	2012	2096 patients after a heart attack	A registry of 19 centres in the US (PREMIER)	Prospective andobservational	One and six months	Rehabilitation programmes	Women were less likely to participate in the programme one month after the start.
[83]	Factors associated with non-attendance at exercise-based cardiac rehabilitation	2019	31,297 patients	The SWEDEHEART national registry, 2010–2016	Observational	Six weeks and one year	Rehabilitation programme	It was more likely that women attended the rehabilitation programme.
[84]	Participation in cardiac rehabilitation, readmissions, and death after acute myocardial infarction	2014	2991 heart attack patients	A single hospital in Minnesota between January 1987 and September 2010	Population-based surveillance study		Rehabilitation programme	1569 (52.5%) participated in rehabilitation; the male participation rate decreased throughout the study.
[85]	Yoga-based cardiac rehabilitation after acute myocardial infarction: A randomized trial	2020	3959 heart attack patients	24 centres in India	Randomised trial	Median: 22 months; minimum: 6 months	Yoga programme (*n* = 1970) compared with traditional treatment plus education (*n* = 1989)	There was no difference in session attendance according to sex.
*Other*								
[23]	Trends in provision of medications and lifestyle counseling in ambulatory settings by gender and race f…	2023	11,033 visits by adults with CHD	Nationally representative ambulatory care, US, 2006–2016	Cross-sectional	In clinic	Lifestyle counselling (e.g., nutrition, exercise, weight reduction)	This was lower in women but not significantly.
[63]	Sex, race, and insurance status differences in hospital treatment and outcomes foll…	2018	38,163 patients after OHCA (42% women)	A Californian regional registry between 2011 and 2015	Retrospective	Five years	Resuscitation	Women had increased odds of receiving a do-not-resuscitate order within one day of admission.
[68]	Age and sex differences and temporal trends in the use of invasive and noninvasive procedures in patients hospi…	2022	1681 men and 1154 women with initial acute MI	All medical centres in central Massachusetts, 2005–2018	Observational	In hospital	Exercise stress testing	There were no sex differences in the receipt of echocardiography and exercise stress testing.
[72]	Evaluation of gender differences in door-to-balloon time in ST-elevation myocardial infarction	2013	735 STEMI patients	2006-2010, two STEMI PCI centres based in tertiary care hospitals in Adelaide.	Prospectively designed registry	In hospital	Timely care provision	Women experienced longer delays in door-to-code time.
[86]	Delayed care and mortality among women and men with myocardial infarction	2017	6022 STEMI patients	An international registry of 41 hospitals between October 2010 and April 2016	Stratified analysis	30 days	Hospital care provision	Despite there being no differences in time from symptom onset to seeking assistance, women experienced longer symptom-onset-to-hospital-arrival times.
[87]	Association of door-in to door-out time with reperfusion delays and outcomes among …	2011	14,821 STEMI patients	298 STEMI units in the US between January 2007 and March 2010	Cohort and retrospective		Referral time	Female sex predicted having a referral time (from hospital arrival to being sent to receive reperfusion) longer than 30 min.
[88]	Sex bias in admission to tertiary-care centres for acute myocardial infarction and cardiogenic shock	2021	340,490 STEMI patients (29.2% female)	Spanish national database, 2003 to 2015	Observational, retrospective study	In hospital	Referral to revascularisation-capable hospital	Women were less frequently admitted to a revascularisation-capable hospital or hospitals with ICCUs than men.
[89]	Gender disparities in patient education provided during patient visits with a diagnosis…	2019	17,332 CVD patients	National survey, the US, 2005–2014	Retrospective, cross-sectional, and observational		Education	Women were less likely than men to receive education targeted towards patients. Female sex was an independent predictor of not receiving education.
[90]	Effect of patient sex on triage for ischaemic heart disease and treatment onset times: A retr…	2014	21,080 MI patients	34 Australian intensive care units between 2005 and 2010	Retrospective		Triage, treatment	More men than women were correctly sent to triage. Time to treatment was shorter in men than in women.
[91]	Social determinants of clinical visits after left main percutaneous coronary intervention versus coronary …	2023	3816 patients after left main PCI or CABG	A single centre, January 2015 to December 2022	Observational	Seven years	Clinic visits	Female gender was a strong predictor of having a follow-up visit.
[92]	Uptake of prescription smoking cessation pharmacotherapies after hospitalization for major cardiovascular disease	2022	20,162 smokers with major CVD (31% female)	All hospitalisations New South Wales, Australia, July 2013–December 2018	Population-based cohort study	90 days post-discharge	Smoking cessation pharmacotherapy (SCP)	Females were more likely than males to be dispensed an SCP prescription; however, this was not maintained after adjusting for potential confounders.

Note: CVD: cardiovascular disease; CHD: coronary heart disease; ACS: acute coronary syndrome; MI: myocardial infarction; PCI: percutaneous coronary intervention; STEMI: ST-elevation myocardial infarction; OHCA: out-of-hospital cardiac arrest; CABG; coronary artery bypass graft; SCP: smoking cessation pharmacotherapy.

**Figure 2:**
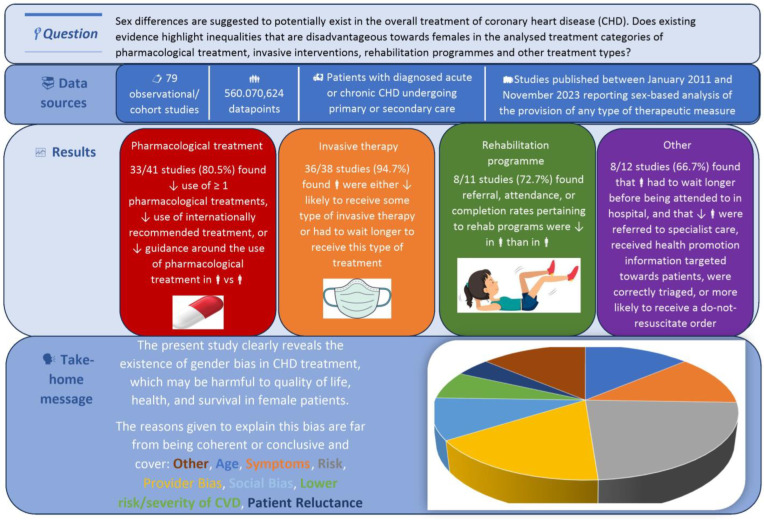
Summary of outcomes uncovered pertaining to gender differences in clinical practice and treatment of coronary heart disease.

In Figure 2, we present a summary of the results of this study.

## 4. Discussion

While it is true that evidence regarding the prevention and treatment of ischaemic heart disease does not differ as a function of sex, the reality is that women receive a different quality and quantity of care than men.

The most effective treatments for preventing and treating CHD are outlined in different clinical practice guidelines established by the European Society of Cardiology (for example, [93,94,95] and the American College of Cardiology and American Heart Association [96], amongst others. These guidelines synthesise and classify current evidence on different treatments of all types in the realm of CHD. Whilst these guidelines highlight important considerations when it comes to applying specific strategies in populations defined by sex, recommendations support the use of each and every strategy indicated with all patients, regardless of sex. Other guidelines regarding CHD treatment recommend the differential use of the most indicated treatments according to sex [97]. In addition, some specific guidelines reiterate that the use of programmes targeting smoking, physical activity engagement, diet, excess body weight, and other risk factors, in addition to pharmacological treatments such as use of beta blockers, ACE inhibitors, anti-lipid agents, aspirin, warfarin and other pharmacological drugs, are also indicated in females [98].

Despite that which is discussed above, the present study demonstrates a clear gender gap that disadvantages females when it comes to receiving treatment for CHD. Further, this gap is evident in all treatment categories examined in the present study. Overall, 80.5% of studies that examined the administration of pharmacological agents to treat CVD identified lower use in women than in men, whilst 94.6% of studies on invasive therapies, 72.7% of studies on rehabilitation programmes, and 66.7% of studies examining other aspects relevant to healthcare provision also concluded that there was poorer provision in women.

A recent study applied meta-analytical procedures to data gathered by 43 studies that analysed prescription prevalence in more than two million patients. The study concluded that women were less likely than men to receive prescriptions for medications recommended to treat CHD, namely aspirin, statins, or ACE inhibitors [99]. The present study reports similar findings and includes other types of medications whilst also considering treatment intensity and duration. It is possible that these differences are associated with perceptions of medical professionals regarding disease severity in women. Currently, chest pain is the predominant symptom for both sexes, but women present a greater number of additional non-chest-pain symptoms, regardless of the presence of chest pain. This may mean that medical professionals are less likely to attribute symptoms in women to heart disease than they are in the case of men [100,101]. Consequently, this reduces the certainty felt by doctors when making a diagnosis, reducing the likelihood of them conducting more in-depth diagnostic tests and administering appropriate and timely treatments [100,102,103].

Generally speaking, rehabilitation programmes are underused with CHD patients. A study conducted by Chan [104] revealed a referral rate of eligible patients of only 18%. This figure is supported by the present findings, which found that only 19% of all included studies examined the use of rehabilitation programmes. Together with the findings of the present review, it is even more concerning that, alongside the general underuse of these programmes, women have even less access to them. In a study conducted by Beckstead [105], after reading 32 hypothetical scenarios, 36 doctors stated whether or not they would refer a hypothetical patient to a rehabilitation programme. Of these doctors, 75% rated women as being less likely to benefit from rehabilitation. Despite this, doctor self-reports about the influence of gender on their decision-making demonstrated that they were not aware of the bias present in their perceptions at the time of deciding whether or not to make a referral. In contrast, doctors reported that perceived patient motivation was the factor that most influenced their decision to make a referral. The present review confirms that the aforementioned gender inequality, shown through hypothetical scenarios, also occurs in practice, although the professionals involved are not conscious of it. Given the importance of having the support of doctors, it is even more concerning that the attitude of professionals could lead to a vicious cycle in which, should doctors not believe the motivation of their female patients, they could cause women to decide not to attend programmes or drop out of them ahead of time, even in cases in which they are referred, because they do not perceive that they have the full support of their doctor [106].

Another relevant finding, aside from the inequalities found with regard to pharmacological treatment, invasive therapies, and rehabilitation programmes, is that other inequalities in other important aspects of healthcare provision emerged. Such aspects included, but were not limited to, waiting time, referral to specialist services, and access to information. This finding may indicate the existence of inequalities in other stages of the chain of healthcare prior to receiving treatment. Research demonstrated that females who suffered a heart attack reported having had prior negative experiences with the healthcare system, such as poor patient–doctor relationships, feeling rejected or not respected, and treatment refusal. These negative experiences influenced later decisions to not seek out healthcare attention [107]. Further, in contrast to that seen with male patients, when females exhibit CHD symptoms and psychological stress, the likelihood of patients being referred to specialist services or receiving treatment in line with established guidelines decreases [108,109].

Potential motives provided within each of the included studies to explain the inequalities identified between males and females were extracted and tabulated. Table 4 presents the conclusions and explanations/justifications made by the included articles. In 71 of the reviewed articles, differences emerged in outcomes that indicated a detriment towards women for some of the aspects studied. Of these, 21% failed to document or explain the causes of these differences, whilst 32% attributed differences to biases in professionals’ practises, 24% associated them with the characteristics and inequalities of the health system, and 23% linked them to biological conditions or the pathological process seen in women.

**Figure 3:**
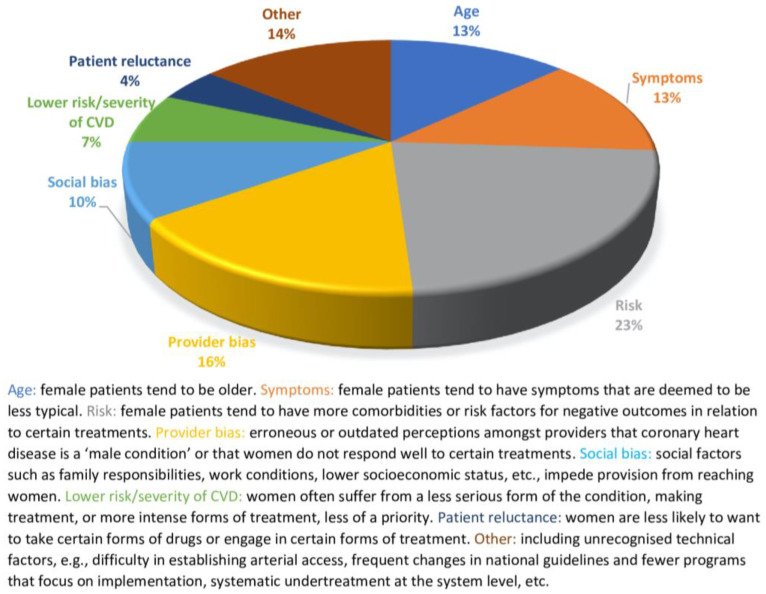
Justifications provided by included studies for identified gender differences in treatment.

The majority of studies surmised potential reasons based on previous research as opposed to directly collecting data on which to ground their conclusions. A number of reasons emerged (Figure 3). First, authors referred to the older age of female participants. This was the case despite the fact that most studies found differences to persist even after controlling for age. Second, the ‘atypical symptoms’ found in women led to their later diagnosis and, ultimately, lesser indication for given treatments. Third, the perceived greater risk of bleeding or mortality related to certain treatments in women was believed to lead to provider reluctance to administer treatment. Fourth, provider bias emerged as a common motive. Fifth, there was social bias, such as lower socioeconomic status, greater responsibilities pertaining to home/family life, etc. Sixth, it is possible that healthcare providers perceive women to be at a lower risk of suffering from severe adverse outcomes from their disease. Finally, a handful of articles surmised that females may be more reluctant to follow certain lines of treatment.

It will not be easy to address these gender biases. This issue is multifactorial in nature and, as such, requires a comprehensive approach which must, first, initiate dialogue with society in order to break down barriers imposed by unhelpful consolidated beliefs. It will be important to work with professionals and to highlight and analyse the shortcomings of clinical practice guidelines. This will take time, perseverance, education, and information.

The recent clinical practice guideline for the treatment of ACS dedicates just half a dozen sentences to raising awareness of this problem. Whilst it begins by describing that ‘there are currently no data supporting the differential management of ACS based on sex’ and briefly outlines some aspects to consider, its consideration of the issue is insufficient. It is possible that a more detailed consideration of the issue placed in a more relevant location of the document could facilitate better understanding of the problem and its implications for patients. Although professionals may already know the content, it is important that they clearly understand its importance and see it reflected in the main guidelines [110].

### Limitations

The reported outcomes of the present review may have been affected by reporting bias in the included articles. It is possible that scientific articles favour the reporting of outcomes which produce significant differences over those which do not, which could lead to gender differences being more likely to emerge in the published literature.

## 5. Conclusions

The present study clearly reveals the existence of gender bias in CHD treatment, which may be harmful to quality of life, health, and survival in female patients. The reasons given to explain this bias are far from being coherent or conclusive. There is an obvious need to find a specific sensitive approach to address the various factors associated with sex and gender [111] as a means of effectively eradicating this gender bias. Thus, in conclusion, it is alarming that 33 years have already passed since the term “Yentl syndrome” was coined to describe prevailing gender bias against women in the handling of coronary disease [112] and, yet, this issue remains as persistent and poorly explained as it was then.

## Figures and Tables

**Table 4:** Conclusions reported by included studies pertaining to gender differences and reasons suggested by study authors for identified gender differences.

Article	Conclusion	Justification
[13]	Male sex was associated with statin treatment. Patients not treated with statins were more likely to be female.	Reasons were undocumented. Possibilities are less medical insurance in females or less likely to have lipid level > 100 mg/dL (with this often being applied as a limit for prescribing statin therapy).
[14]	Fewer women were treated with thienopyridines, heparin, and glycoprotein IIb/IIIa inhibitors compared to men. Female gender was independently associated with a lower in-hospital use of coronary angiography.	The underestimation of patient risk was the most common reason for not pursuing an invasive strategy in both men and women.
[15]	After adjusting for covariates, males had higher odds of statin prescription in coronary heart disease patients.	Not documented.
[16]	Eligible women were less likely to receive evidence-based acute treatments, rehabilitation, and reperfusion treatments.	Women were presented to hospitals for STEMI treatment 1.4 h later than men. Though analysis was conducted on eligible patients with treatment indications and adjusted for clinical factors, women were older and had worse clinical profiles than men. Worse clinical profiles contribute to an increased risk of adverse outcomes which are associated with undertreatment, partly for fear of complications (e.g., higher risk of bleeding) and partly because of the lack of evidence from randomised clinical trials in these groups.
[17]	Long-term statin prescriptions were less common in women.	Female gender has been shown to be a risk factor for statin-induced myalgias, which could lead to early discontinuation. Intense media publicity of exaggerated side effects of statins may have a negative impact on the continuation of statins, with more profound effects on women.
[18]	Factors associated with filling high-intensity statin prescriptions included male sex.	This may reflect variation in the use of evidence-based therapies across hospitals and providers or the use of intensive medical management for select high-risk patients.
[19]	Male sex was associated with a greater likelihood of statin utilisation.	Not documented.
[20]	Females were less likely to receive PCI and CABG.	Stark differences in treatment between males and females reflected an excess risk of readmission and mortality following an MI in males.
[21]	Revascularisation treatment, prescriptions of platelet aggregation inhibitors, statins, and functional rehabilitation were more frequently provided to men than women.	Women were less likely to have multiple injuries than men, which might explain (at least in part) sex differences in reperfusion therapy. Studies have suggested that a higher haemorrhagic risk and the presence of relative contraindications could explain the difference in the prescription of platelet aggregation inhibitors. The lack of the universality of the health system and of a clearly specified care pathway for ACS is a baseless argument for gender differences based on what is found here.
[22]	Among patients without contraindications, women were less likely to receive treatments than men. In particular, we found that the use of invasive strategies (which included PC) was lower in women.	Women less often presented with chest discomfort than men. Lower eGFR levels in women than men may also partly explain sex differences. The reasons for women less often undergoing an invasive strategy may relate to the differences in baseline characteristics and physician recognition of NSTEMI in women.
[23]	Women were less likely than men to receive medications for secondary prevention.	There are frequent changes in national guidelines and fewer programmes that focus on implementation.
[24]	Characteristics associated with lower odds of statin therapy initiation included female sex.	This may be due to misperceptions of adverse effects of statins and uncertainty among both patients and prescribers about statin efficacy in women and older patients.
[25]	During hospital admission for an ACS, the proportion of women that used cardiovascular drugs was lower than that of men.	There were differences in the severity of the ACS and comorbidities and differences in the prescription attitude of physicians or differences in attitude to taking the drugs by patients.
[26]	The use of all five indicated drugs and PCI was higher in male patients compared to females.	Not documented. Generally, symptom patterns are more atypical in women, making it more difficult to diagnose cardiac pathology. Women are considered at a lower risk of acute MI, which makes physicians less aware of the risk of new cardiovascular events, causing lower medical adherence. Once acute MI has occurred, young women are at a high risk of complications. Also, trials have enrolled few women; however, benefits concerning safety and outcomes have been observed regardless of gender.
[27]	Male sex was related to the prescription of statins at discharge.	Not documented.
[28]	Women were less likely to either have initiated statin therapy or to have persistent statin therapy at the end of follow-up.	The older age at CAD onset in women may have contributed to the sex disparities in statin therapy. Women were less likely to be evaluated by a cardiologist, which may have contributed to sex disparity in statin use given that cardiologist evaluation contributed to statin use in general. Differences in reported adverse events to statin treatment was a significant contributor to the sex disparity in statin therapy.
[29]	Women were less likely to be prescribed statins and antiplatelet drugs.	It is impossible to judge from our data whether this difference is due to physicians taking a different attitude to prescribing statins for the two genders or to men and women responding differently to the offer of statin treatment.
[30]	Women were less likely to be discharged with aspirin and clopidogrel.	Women with an ACS diagnosis are more likely to be older than men and to have diabetes, hypertension, heart failure, or other comorbidities.
[31]	Proportionally fewer female patients underwent invasive procedures.	Findings show the possibility that health inequality in female patients with AMI arises from social inequality rather than biological differences such as age and severity. Females severely trail males in economic participation and opportunity, educational attainment, health and survival, and political empowerment. Furthermore, the cause of myocardial infarction in females is less well understood than in males. Also, most of the information and educational materials available to the public focus on AMI symptoms in males.
[32]	Factors associated in multivariable analysis with suboptimal statin occurrence included female sex.	Not documented.
[33]	Selected patient factors associated with the non-use of high-potency statins included female sex.	The study highlights the need to educate physicians and mentions reluctance to treating high-risk patients with a high-intensity regimen.
[34]	Women with STEMI were less likely to have undergone coronary angiography or revascularisation and have received timely revascularisation or primary PCI and less likely to receive some elements of secondary preventive treatment, including b-blockers and statins, and referral to cardiac rehabilitation.	Women presented with ACS later in life than men and had more comorbid conditions, especially diabetes and hypertension, but women with STEMI in every GRACE risk score quartile received less comprehensive. assessment and treatment. Undertreatment in the population examined might have been caused by poor awareness that women with STEMI are generally at a higher risk or by a preference for subjectively determining risk rather than applying more reliable, objective risk prediction tools.
[35]	PCI and DES were more often used in males. Males were more often treated with dual antiplatelet therapy.	Not documented. Generally, there is a perception of the risk of bleeding.
[36]	Women were less likely to be prescribed evidence-based treatment at discharge (b-blockers, angiotensin-converting enzyme inhibitors, or angiotensin receptor blockers, statins, and P2y12 antagonists). Also, women with STEMI were less likely than men to undergo coronary angiography and PCI.	There is systematic undertreatment. We cannot rule out that findings reflect a sexist bias. Effective measures within the healthcare system are needed to neutralise the undertreatment of women after MI.
[37]	Female sex was a predictor of the incomplete prescription of guideline medications at discharge.	The disparity between men and women occur mainly with the prescription of statins, with women receiving medium- or low-intensity statin therapy more often than men. Reasons for this are yet to be elucidated, with the hypothesis that there is a perceived risk for adverse outcomes after. ACS is lower in young women because of a traditional school of thought dictating that cardiovascular disease is primarily a male disease.
[38]	Women had lower odds of attending cardiac rehabilitation and using secondary prevention medications.	There was a combination of financial, social, and psychological reasons. Lower socioeconomic status in women may have led to difficulties in adhering to secondary prevention care due to the out-of-pocket cost.
[39]	In the dual-high risk group, females were less likely to receive guideline-recommended therapy.	Traditionally, lower rates of invasive management in females have been attributed to differences in baseline risk for further bleeding and ischemia compared to males. This is likely due to physicians’ preference for a conservative management strategy in this group to avoid commitment to long-term DAPT in the case of PCI, although there is emerging evidence to support short-term DAPT in high-bleeding-risk patients, regardless of the complexity of their PCI procedure. However, these concerns do not justify differences in [the receipt of guideline-recommended therapy between sexes of the same combined risk group. Guidelines do not provide recommendations on how patients at a high risk of both ischemic and major bleeding complications should be managed.
[40]	Younger men were significantly more likely than younger women to initiate appropriate treatment. There was no significant sex difference in adherence to medication therapy.	Cardiovascular disease among younger women has only recently received research attention. Perceptions of risk for adverse outcomes in physicians and patients is still skewed for younger women, who are seen as healthy and at low risk.
[41]	Female gender was associated with a higher probability of a switch to a less effective statin.	In all cases, the decision to switch to a less intensive lipid-lowering intervention was taken by primary care physicians, either following the occurrence of side effects or for safety concerns.
[42]	A lower proportion of women received high-potency antiplatelet agents.	There were greater hemorrhagic risk scores among women. Several relative contraindications to high-potency antiplatelet drugs, such as advanced age, hypertension, and small body size, were more common among women.
[43]	Female sex predicted the discontinuation of DAPT.	The duration of DAPT may have been shortened during the early treatment phase because of concerns regarding the risk of bleeding.
[44]	In adjusted models, female gender was independently associated with a lower likelihood of receiving statins or high-intensity statins.	Our results do not explain why clinicians are less likely to use statins or high-intensity statins in female patients with CVD. This could reflect providers’ perception of a lower risk of recurrent CVD events in female patients with CVD despite results showing that women have a higher overall illness burden. The lower use of statins and high-intensity statins in female patients with CVD may also represent healthcare providers’ perception of a lower benefit from statins in females. Findings also fail to explain whether patient preferences or differences in the side effects of high-intensity statins explain sex differences. However, it is known that the side effects of these druges do not differ according to sex.
[45]	Independent predictors of any prasugrel cessation included female sex.	Female sex previously emerged as an independent predictor of bleeding in prasugrel-treated patients. The leading cause of prasugrel disruption (in all patients) was cessation because of unjustified patient decision.
[46]	After adjustment, lipid-lowering drugs, beta blockers, clopidogrel, and aspirin were all redeemed equally.	
[47]	There was no difference in pharmaceutical use but more men were treated with thrombolysis.	It may be because of the earlier recognition of MI and less distractions to reaching hospitals.
[48]	No important sex differences.	Results may indicate increased awareness that women have adverse outcomes similar to and, in some cases, worse than men and also the recognition that females have higher rates of hypertension and/or microvascular disease or coronary vasospasm underlying their stable angina.
[49]	Compared to men, women were 10 years older, had more comorbidities, less often underwent angiography and reperfusion, and received less medical treatment.	The less frequent use of invasive procedures in women was not only related to age and baseline characteristics but also reflected systematic undertreatment.
[50]	Women were less likely to receive statins. Overall, no gender differences were found in the total daily dose intake. Women were less likely to undergo revascularisation.	A possible explanation could be that women were less likely to adhere to statins because of more side effects. However, no gender differences were observed in self-reported medication adherence, statin intolerance, or patient refusal to take statins. It is possible that women did not require a revascularisation procedure because of the presentation and severity of the underlying disease.
[51]	Some drugs were favoured in men and others were favoured in women.	Findings do not imply ‘inequality’, which may be due to the fact that existing guidelines are based on trials with a higher percentage of men. Women may be more vulnerable to the adverse effects of drug therapy, and disease-specific guidelines do not take into account sex differences in ACS patients.
[52]	Women were less likely to receive reperfusion and early invasive therapies.	Women with or without prior CABG were older and had a higher risk profile than men. Women with prior CABG need to be more represented in studies.
[53]	After controlling for demographics and comorbidities, ethnic minorities and women had lower rates of revascularisation compared to white men.	Sex bias in use of aggressive treatment may play a role.
[54]	Reperfusion therapy was used less often in women.	In older women, there was disparate use often explained by adjusting for age and associated comorbidities in women; however, even after controlling for these factors, sex disparities persisted, which may suggest a biassed management of older women. In younger women, disparities were more difficult to explain, the most explanatory factor was the recognition of atypical symptoms.
[55]	After adjustment for age, comorbidity, and hospital characteristics, women had a lower probability of undergoing revascularisation procedures.	Women may present with “atypical” symptoms and physicians’ recommendations for cardiac catheterisation may also differ. Rather than lower access to effective treatment for women, we might be witnessing the overuse of revascularisation procedures in men.
[56]	Female sex was an independent variable associated with no-reperfusion therapy.	Not documented.
[57]	Both younger and older women received less frequent angioplasty with stents and more often received no reperfusion treatment than men in corresponding age groups.	There was an under-investigation of older women who were the largest group. A higher proportion of younger women in this study lived in rural areas than younger men and fewer older women were English-speaking compared to their older male cohort.
[58]	Rates of PCI in the CTO group were similar between genders; however, women with CTO were treated significantly less by CABG compared to men. Moreover, compared to male patients, significantly fewer women undergoing CABG had the revascularisation of the CTO artery.	Although female gender was an independent predictor of medical management, other adverse characteristics, including older age and higher comorbidities, were likely contributing factors. It is also possible that smaller distal vessels may be more common in women with CTOs.
[59]	Patients were less likely to receive revascularisation if they were female.	Not documented.
[60]	A factor associated with not attempting reperfusion therapy was female sex.	Recent data indicate that women are more likely to exceed in-hospital and pre-hospital time guidelines for p-PCI with consequent higher chance of missing the opportunity for reperfusion.
[61]	Although women had smaller vessel sizes, they received DES less often compared to men.	There was an intention to prevent bleeding complications in women by the use of BMS instead of DES.
[62]	In a real-life setting, women with STEMI/NC ACS were less likely than men to be managed invasively.	Not documented. Generally, factors related to healthcare systems (delay between symptom onset and seeking help) and patients (e.g., women’s own preferences) influence ability to perceive, seek, reach, pay for, and engage in healthcare and the ability of the system to fulfil patient needs.
[63]	Female sex was independently associated with lower rates of treatment at a 24/7 PCI centre and lower rates of cardiac catheterisation.	Given that their survival with good neurological outcomes is only slightly worse, interventional cardiologists may appropriately select women who are most likely to benefit from cardiac catheterisation. Female patients may manifest more atypical symptoms than men preceding OHCA, leading to the delayed recognition of cardiac aetiology and delayed transfer to a 24/7 PCI centre.
[64]	Female sex was an independent factor for not undergoing PCI.	Less serious cardiac events were probable in women.
[65]	Women were less likely to receive an indicated therapy prior to hospitalisation and to receive an invasive intervention upon arrival. Fewer women received angiography or PCI and an indicated therapy following MI.	Differences in admission route due to greater diagnostic uncertainty amongst women reduces the chance of revascularisation. Delays in time between symptom onset and first contact with medical services mean that the benefits of emergent coronary revascularisation are less certain. Emergency care decisions regarding coronary angiography and PCI in women may be influenced by smaller coronary anatomy, more technically challenging vascular access (the excess door-to-balloon time seen in older women in this study may reflect this), and a greater risk of procedure-related complications and post-procedural mortality.
[66]	Younger women with STEMI were more likely to seek acute medical care and less likely to receive thrombolytic therapies or primary PCI and guideline-recommended pharmacotherapy than men.	Less than 50% of women received an ECG within ten minutes, suggesting that sex differences exist in the diagnosis of STEMI, possibly due to the poor recognition of atypical symptoms.
[67]	Women were less likely to undergo angiography or PCI.	There was a notion that women suffer worse outcomes, though this was found to not be true.
[68]	A smaller proportion of women underwent cardiac catheterisation, percutaneous coronary intervention, and coronary artery bypass graft surgery.	A lack of typical chest pain and delays in seeking acute medical care among women may not result in indications for the receipt of coronary angiography or coronary reperfusion therapy, which are symptom- and time-dependent. This would explain why no sex differences were found in use of echocardiography or exercise stress testing, as these procedures do not depend on the presence of chest pain symptoms.
[69]	Women experienced longer call-to-hospital-arrival time and were less likely to receive DAPT, beta blockers, ACE inhibitors, coronary angiography, and PCI. After adjustment, women had significantly lower odds of receiving coronary angiography and CABG.	Not documented. Generally, unconscious bias among physicians and higher mortality rates in women act as competing risk factors. There is hesitancy to expose women to provide resuscitation.
[70]	There was sex disparity in diagnostic angiography, PCI, and CABG use favouring men.	There are sex-based biological differences.
[71]	Second-generation DES use was significantly less frequent among women.	Differences came from the first 1.5 years of the study when there was less awareness of benefits in women.
[72]	Upon hospital arrival, women experienced delays in door-to-code, code-to-balloon, and thus DTB time. After multivariate adjustment, independent determinants of DTB time included female gender.	Women tended to have less peak ST-elevation than men in this study and this may have contributed to delayed ECG interpretation. LTB time was significantly delayed in women, which may indicate that unrecognised technical factors contribute to delayed DTB time. These may include difficulty in establishing arterial access, the catheter engagement of the infarct-related artery, the passage of the guidewire/balloon through the coronary occlusion, or even balloon inflation itself.
[74]	Median system delay and revascularisation delay were longer in women.	Not documented. Generally, later diagnosis due to atypical symptoms or higher prevalence of acute coronary syndrome without obstructive coronary disease, suggesting negative discrimination by the healthcare system.
[75]	There were longer median times in females and females were less likely to receive FMC-to-ballon times of less than 90 mins. There were significantly longer total and direct STB, FMCTB, and DTB durations for females. There were significant differences in geometric mean times from symptom onset to FMC.	A greater proportion of females presented after 3 h from symptom onset. There was variability in symptoms between men and women, with women experiencing a wider variety and less common symptoms occurring more often. There were lower rates of initial successful radial arterial approaches in females.
[76]	Females were more likely to receive an inappropriate treatment.	Not documented.
[77]	Women were more likely to undergo DES implantation.	Not documented.
[78]	System delay was unaffected by sex in the multivariate model.	
[79]	Male gender was associated with significant increased referral to rehabilitation in all hospitals.	Differences were concentrated in the lowest-referring hospitals with the lowest-referring hospitals serving proportionally more female and minority patients than the highest-referring hospitals.
[80]	Non-referral was independently associated with female sex.	Limited capacity at CR facilities or a lack of reimbursement for specific subgroups may also lead to lower referral rates. Also, studies have suggested that some physicians believe that women benefit less from CR.
[81]	Subjects who completed CR were more likely to be male.	Socioeconomic status and lower levels of education have been associated with poor attendance.
[82]	Females were less likely to participate in cardiac rehabilitation at 1 month.	Studies have suggested that women are more likely to have barriers related to transportation, family responsibilities, comorbidities, and perceiving exercise as tiring.
[83]	Male sex was a predictor of non-attendance.	Sweden is one of the world’s most gender-equal countries regarding typical barriers to women (family obligations, caretaking responsibilities, and multiple role conflicts).
[84]	Participation in rehab decreased in men but not in women over the study period.	Not documented.
[85]	There was no difference in attendance at Yoga-CaRe sessions by sex.	
[86]	Despite similar delays in seeking care, the overall time from symptom onset to hospital presentation was longer for women than men.	Differential treatment and triage strategies used by first responders and transfer procedures between hospitals. Longer delays on the part of the health system than on the part of patients, most likely a delay in diagnosis by the provider.
[87]	Significant factors associated with a DIDO time greater than 30 min included female sex.	The study states that the magnitude of this contribution of sex was small and did not discuss it further.
[88]	Women were less often admitted to revascularisation-capable hospitals than men.	Age played an important role in admission to a revascularisation capable hospital or ICCU centre.
[89]	Females had 0.86 times the odds of receiving patient education compared to males.	This may have been partially attributable to clinicians following the USPSTF guidelines for lipid screening that were less aggressive for females.
[90]	Proportionately more men were triaged correctly for AMI than women. Across all triage categories, average treatment time was faster for men than women. When incorrectly triaged, treatment time for men was faster than for women.	A correct triage score was vital for treatment time and more women were incorrectly triaged.
[91]	Female gender was a strong predictor of having a follow-up visit.	Not discussed regarding sex. Structural barriers to healthcare access were discussed generally.
[92]	Females were more likely than males to be dispensed a prescription smoking cessation programme (SCP).	Sex difference did not persist after adjustment for mood disorders and prior use of prescription SCP.

Note: CVD: cardiovascular disease; CHD: coronary heart disease; ACS: acute coronary syndrome; MI: myocardial infarction; PCI: percutaneous coronary intervention; STEMI: ST-elevation myocardial infarction; NSTEMI: non-ST-elevation myocardial infarction; ACE: angiotensin-converting enzyme; OHCA: out-of-hospital cardiac arrest; CABG; coronary artery bypass graft; DES: drug-eluting stent; SCP: smoking cessation pharmacotherapy.

## Data Availability

No new data were created or analyzed in this study. Data sharing is not applicable to this article.

## Supplementary Materials

The following supporting information can be downloaded at https://www.mdpi.com/article/10.3390/jcm14051583/s1. Table S1: Study quality of multiple-measure cohort studies assessed using Newcastle–Ottawa Quality Index; Table S2: Study quality of cross-sectional, single-measure cohort or similar studies assessed using Newcastle–Ottawa Quality Index.

## Abbreviations

The following abbreviations are used in this manuscript:

CHD	coronary heart disease
CVD	cardiovascular disease
ACE	angiotensin-converting enzyme
PCI	percutaneous coronary intervention
DAPT	dual antiplatelet therapy
STEMI	ST-segment-elevation myocardial infarction
