# Supplementary material for: Gender Differences in Clinical Practice Regarding Coronary Heart Disease: A Systematic Review
Source: J Clin Med. 2025 Feb 26;14(5):1583. doi: 10.3390/jcm14051583 (PMC11900247; doi:10.3390/jcm14051583)
Supplement: Supplementary file 1 [file jcm-14-01583-s001.zip › Supplementary Materials.pdf]

Supplementary Table S1. Study quality of multiple measure cohort studies assessed using the Newcastle-Ottawa Quality Index.

	Selection				Comparability	Results			Classification
**Article**	**1**	**2**	**3**	**4**		**1**	**2**	**3**	
Arslan et al. 2021	1	1	1	1	0	1	1	1	Low
Borg et al. 2019	1	1	1	1	2	0	1	1	High
Brouwers et al. 2021	0	1	1	1	2	1	1	1	High
Bruggmann et al. 2020	0	1	1	1	2	0	1	1	High
Buja et al. 2014	1	1	1	1	1	1	1	0	High
Chou et al. 2018	1	1	1	1	0	1	1	1	Low
Cirillo et al. 2019	1	1	1	1	1	1	1	1	High
Colbert et al. 2015	1	1	1	1	0	1	1	1	Low
Colivicchi et al. 2011	0	1	1	1	2	1	1	1	High
Dankner et al. 2015	0	1	1	0	2	0	1	1	Average
Dunlay et al. 2014	1	1	1	1	1	1	1	0	High
Eindhoven et al. 2018	1	1	1	1	1	1	1	1	High
Eisen et al. 2017	1	1	1	1	2	1	1	1	High
El Missiri et al. 2020	0	1	1	1	0	1	1	1	Low
Farkowski et al. 2014	0	1	1	0	0	0	1	0	Low
Galway et al. 2017	1	1	1	1	2	1	1	1	High
Gravely et al. 2014	1	1	1	0	0	0	1	0	Low
Guimaraes et al. 2017	1	1	1	1	0	0	1	1	Low
Hoedemaker et al. 2021	0	1	1	1	2	0	1	0	Low
Hojskov et al. 2020	0	1	1	1	1	0	1	0	Low
Hoo et al. 2016	0	1	1	0	2	0	1	0	Low
Huber et al. 2019	1	1	1	0	2	0	1	0	Low
Hyun et al. 2021	1	1	1	0	1	1	1	1	High
Jackson et al. 2020	1	1	1	1	2	1	1	1	High
Jankowski et al. 2021	1	1	1	0	2	0	1	1	High
Jimeno-Sanchez et al. 2019	0	1	1	1	0	1	0	1	Low

Kim y So. 2019	1	1	1	0	0	0	1	0	Low
Koskinas et al. 2018	1	1	1	1	2	0	1	1	High
Kummer et al. 2015	1	1	1	1	2	1	1	1	High
Lin et al. 2014	1	1	1	1	2	1	1	1	High
Martin et al. 2012	1	1	1	1	2	1	1	0	High
Mommersteeg et al. 2021	1	1	1	0	0	0	1	1	Low
Nakamura et al. 2020	1	1	1	1	2	1	1	1	High
Parashar et al. 2012	1	1	1	0	2	0	1	1	High
Perera et al. 2021	0	1	1	0	0	0	1	0	Low
Prabhakaran et al. 2020	1	1	1	1	1	0	1	1	High
Proença et al. 2023	0	1	1	1	0	0	1	0	Low
Ruiz-Pizarro et al. 2019	0	1	1	1	1	0	1	1	High
Setny et al. 2021	1	1	1	0	0	0	1	0	Low
Smolina et al. 2015	1	1	1	0	2	1	1	1	High
Thalmann et al. 2022	1	1	1	0	2	1	1	1	High
Tsui et al. 2012	1	1	1	1	0	0	1	0	Low
Turner et al. 2017	1	1	1	0	2	1	1	1	High
Udell et al. 2017	1	1	1	0	2	0	1	0	Low
Vynckier et al. 2020	1	1	1	1	2	0	1	1	High
Yu et al. 2016	1	1	1	1	0	0	1	1	Low
Zandecki et al. 2017	1	1	1	1	0	1	1	1	Low
Zhang et al. 2016	1	1	1	0	2	1	1	1	High

Supplementary Table S2. Study quality of cross-sectional, single measure cohort or similar studies assessed using the Newcastle-Ottawa Quality Index.

	Selection				Comparability	Results		Classification
**Article**	**1**	**2**	**3**	**4**		**1**	**2**	
Al-Aqeedi et al. 2012	1	1	1	2	2	2	1	High
Ambrosino et al. 2023	1	1	1	2	0	2	0	Low
Aragam et al. 2011	1	1	1	2	2	2	0	High
Araújo et al. 2018	1	1	1	2	2	2	0	High
Arias-Mendoza et al. 2023	1	1	1	2	0	2	0	Low
Arnold et al. 2011	1	1	1	2	2	2	1	High
Ashfaq et al. 2023	1	1	0	2	2	2	1	High
Ashraf et al. 2023	1	1	1	2	2	2	1	High
Baber et al. 2016	1	1	1	2	2	2	0	High
Bahall et al. 2019	1	1	1	1	2	1	1	High
Berlin et al. 2016	1	1	1	1	2	2	0	High
Bhasin et al. 2023	1	1	1	2	2	2	1	High
Bhatt et al. 2015	0	0	1	1	0	2	0	Low
Bouisset et al. 2021	1	1	0	1	2	2	0	Average
Bugiardini et al. 2017	1	1	1	2	1	2	1	High
Burgess et al. 2023	1	1	1	2	0	2	0	Low
Cabrerizo-Garcia et al. 2015	0	0	1	1	0	1	0	Low
Calé et al. 2019	1	0	1	2	2	1	1	High
Carbajosa Dalmau et al. 2011	0	0	1	0	0	0	1	Low
Cenko et al. 2016	1	1	1	2	2	2	1	High
Chatterjee et al. 2017	0	1	1	2	0	2	0	Low
Chen et al. 2022	1	1	1	2	0	2	0	Low
Chen et al. 2018	1	1	1	2	0	2	0	Low
Collado-Lledó et al. 2020	1	0	1	2	0	1	0	Low
Corrada et al. 2014	1	1	1	1	1	2	0	Average
Dafaala et al. 2022	1	1	1	1	2	2	1	High

De Matos Soeiro et al. 2018	1	0	1	2	0	1	0	Low
Dillinger et al. 2021	1	1	0	0	0	0	0	Low
Dreyer et al. 2013	1	1	0	2	2	2	1	High
Ebbinghaus et al. 2012	1	0	1	2	0	1	0	Low
Ferrari et al. 2013	1	1	1	2	0	2	0	Low
Ferraz-Torres et al. 2015	0	1	1	2	2	0	1	Low
Ferraz-Torres et al. 2014	0	1	1	2	0	0	0	Low
Flores-Umanzor et al. 2020	0	0	1	2	0	1	0	Low
Ford et al. 2020	0	1	1	2	0	2	0	Low
Freund et al. 2012	0	1	1	2	2	1	1	High
Gauthier et al. 2022	1	1	0	2	2	2	0	High
Ghadri et al. 2015	1	0	0	2	0	2	0	Low
Ghauharali-Imami et al. 2015	0	0	0	2	0	2	0	Low
Gijón-Conde y Banegas. 2011	1	1	0	2	0	2	0	Low
Gnavi et al. 2014	1	1	1	2	1	2	1	High
Guo et al. 2022	1	1	0	2	1	2	0	High
Hansen et al. 2012	1	1	1	2	2	2	1	High
Hao et al. 2019	1	1	1	2	2	2	1	High
Hersi et al. 2013	1	0	0	2	0	2	0	Low
Hess et al. 2014	1	1	0	2	0	2	0	Low
Hilleary et al. 2019	1	1	0	2	2	1	1	High
Hollanda Oliveira et al. 2022	1	0	0	2	2	1	0	Low
Hong y Kang. 2015	1	1	0	2	2	2	1	High
Janion-Sadowska et al. 2011	0	0	1	2	0	2	0	Low
Jegier et al. 2011	0	0	0	2	1	0	0	Low
Jimeno-Sanchez et al. 2019	0	0	1	2	0	2	0	Low
Jin et al. 2014	0	0	0	2	2	1	1	Low
Johnston et al. 2013	1	1	1	2	2	2	1	High
Josiah y Farshid. 2019	1	1	0	2	0	1	0	Low
Kalra et al. 2016	1	1	1	2	0	2	0	Low

Kerkman et al. 2020	1	0	0	2	0	2	0	Low
Khan et al. 2018	1	1	0	2	1	2	1	High
Kislitsina et al. 2019	0	0	0	2	0	0	0	Low
Koçyigit et al. 2018	1	0	1	2	0	1	0	Low
Komajda et al. 2021	1	0	0	2	2	2	0	Average
Koopman et al. 2013	1	1	1	2	2	2	0	High
Kotseva et al. 2012	1	1	0	2	2	1	0	Average
Kragholm et al. 2015	1	1	0	2	0	0	0	Low
Kristic et al. 2021	0	0	0	2	0	2	0	Low
Kuehnemund et al. 2023	1	1	1	2	0	2	0	Low
Kuhn et al. 2015	1	1	1	2	1	2	0	High
Kuhn et al. 2014	1	1	1	2	1	2	0	High
Kytö et al. 2019	1	1	0	2	2	2	1	High
Lee et al. 2021	1	1	1	2	0	2	0	Low
Lee et al. 2019	1	1	0	2	2	1	0	Low
Leurent et al. 2014	1	1	1	2	2	0	1	Low
Li et al. 2023	1	1	1	2	0	2	0	Low
Li et al. 2018	1	1	1	2	0	2	0	Low
Lin et al. 2013	1	0	1	2	0	2	0	Low
Long et al. 2020	0	0	0	2	0	0	0	Low
Magee et al. 2015	1	0	0	2	0	1	0	Low
Mahajan et al. 2017	1	0	0	2	0	1	0	Low
Mazurek et al. 2018	1	1	0	2	2	1	0	Low
Mehta et al. 2011	1	1	1	2	2	1	0	Low
Meyer et al. 2019	1	1	1	2	2	2	1	High
Minhas et al. 2022	1	1	1	2	2	2	1	High
Mirghani et al. 2016	0	0	0	2	0	2	0	Low
Minten et al. 2023	0	0	1	2	0	0	0	Low
Mohamed et al. 2021	1	1	1	2	2	2	1	High
Moradi et al. 2011	0	0	0	0	0	0	0	Low

Morton et al. 2022	1	1	1	2	2	2	0	High
Mufarreh et al. 2023	1	1	1	2	2	2	0	High
Murphy et al. 2019	1	1	1	2	0	2	0	Low
Musa et al. 2019	0	0	1	2	1	2	0	Average
Myftiu et al. 2015	1	0	0	2	2	2	0	Average
Naito et al. 2016	0	1	0	2	0	1	0	Low
Nalini et al. 2014	1	1	1	2	0	2	0	Low
Ngo-Metzger et al. 2019	1	1	0	1	2	1	1	Average
Nguyen et al. 2014	0	0	1	2	0	2	0	Low
Olivencia Peña et al. 2011	0	0	0	2	0	0	0	Low
Pagidipati et al. 2013	1	0	0	2	2	1	1	Average
Pandey et al. 2016	1	1	0	2	2	2	1	High
Patel et al. 2015	1	1	1	2	0	2	0	Low
Pereira et al. 2014	1	0	1	2	1	2	0	High
Piatek et al. 2018	1	1	1	2	0	1	0	Low
Plaza-Martín et al. 2019	1	0	1	2	2	0	1	Low
Poon et al. 2012	1	1	0	2	2	2	1	High
Pouncey et al. 2022	0	1	1	2	0	2	0	Low
Redfors et al. 2015	1	1	1	2	2	1	1	High
Riehle et al. 2023	1	1	1	2	0	2	0	Low
Riesgo et al. 2011	1	0	0	2	2	1	0	Low
Robijn et al. 2022	0	1	1	2	2	2	0	High
Roe et al. 2013	1	0	0	2	2	2	1	Average
Romero et al. 2018	1	0	0	2	2	2	1	Average
Ronco et al. 2023	1	0	0	2	2	1	0	Average
Roque et al. 2020	1	1	1	2	0	1	1	Low
Rosenson et al. 2017	1	1	0	2	2	2	1	High
Russ et al. 2017	1	1	1	2	2	2	1	High
Sadowski et al. 2013	1	0	1	2	0	2	0	Low
Sakata et al. 2014	1	1	0	2	0	1	0	Low

Sambola et al. 2021	1	1	1	2	2	2	0	High
Schiele et al. 2011	1	1	0	2	1	2	0	High
Shehab et al. 2020	1	1	1	2	2	2	1	High
Sheppard et al. 2012	1	1	1	2	1	2	0	Average
Siabani et al. 2020	0	0	0	2	0	2	0	Low
Singh et al. 2013	1	0	0	2	0	0	0	Low
Sobti et al. 2022	1	1	1	2	0	1	0	Low
Soholm et al. 2021	0	0	0	2	1	2	1	Low
Sulaiman et al. 2021	1	1	1	2	2	2	1	High
Sulaiman et al. 2011	1	0	1	2	0	2	0	Low
Sundaram et al. 2020	1	1	1	2	2	2	0	High
Taha et al. 2012	0	0	1	2	0	0	0	Low
Tang et al. 2021	1	1	1	2	0	2	0	Low
Tran et al. 2022	1	0	1	2	2	2	0	High
Tran et al. 2017	1	0	0	1	1	1	1	Low
Trninic et al. 2014	1	0	1	2	2	0	1	High
van Engen-Verheul et al. 2012	1	1	1	2	0	2	1	Low
Varma et al. 2023	1	1	1	2	0	2	1	Low
Vatcheva et al. 2019	1	1	1	2	2	2	1	High
Victor et al. 2014	1	1	1	2	2	0	1	Low
Virani et al. 2015	1	1	1	2	2	2	0	High
Vogel et al. 2016	0	0	1	2	2	2	1	Average
Wang et al. 2022	1	1	1	2	0	2	0	Low
Wang et al. 2011	1	1	1	2	2	2	1	High
Whittle et al 2022	0	0	0	2	0	2	1	Low
Wolff et al. 2016	1	1	1	2	2	1	1	High
Worrall-Carter et al. 2017	0	0	0	2	2	0	1	Low
Xanthopoulou et al. 2017	1	0	1	2	0	2	0	Low
Yu et al. 2015	1	1	1	2	0	2	1	Low

Yu et al. 2011	1	1	1	2	0	1	0	Low
Yusniawati et al. 2020	0	0	0	2	2	2	1	Low
Zheng et al. 2019	1	1	1	2	0	2	1	Low
